# Start-up costs of implementing Screening and Brief Intervention (SBI) for Adolescents (part of Economics of SBI symposium)

**DOI:** 10.1186/1940-0640-10-S2-O5

**Published:** 2015-09-24

**Authors:** Carolina Barbosa, Laura J Dunlap, Brendan Wedehase, Shannon Gwin Mitchell, Robert P Schwartz, Kristi Dusek, Arethusa S Kirk, Marla Oros, Colleen Hosler, Jan Gryczynski, Barry S Brown

**Affiliations:** 1 RTI International, Chicago, Illinois, USA; 2 RTI International, Research Triangle Park, North Carolina, USA; 3 Friends Research Institute, Baltimore, Maryland, USA; 4 Total Health Care, Baltimore, Maryland, USA; 5 Mosaic Group, Baltimore, Maryland, USA; 6 University of North Carolina at Wilmington, Wilmington, North Carolina, USA

## Background

Understanding the costs to implement SBI is important for providers in planning resource needs, and for decision makers considering widespread implementation of SBI. Unfortunately, little is known about the initial costs to start an SBI program.

The objective is to estimate the start-up costs of two models of SBI delivery to adolescents in primary health care settings: BI delivered by a behavioral health specialist (specialist model) and BI delivered by a primary care provider (generalist model).

## Materials and methods

SBI was implemented in a multi-site, cluster randomized trial (N = 7) guided by Proctor's model of implementation. The economic costs of starting SBI were calculated using an activity-based costing methodology. Data collection instruments were developed to collect staff time spent in identified SBI activities and non-labor resources. Start-up activities included: 1) administrative activities, such as changes to existing electronic medical record systems and planning meetings; 2) staff training; and 3) technical assistance.

## Results

The average total cost for initial implementation of SBI was $5,017 and $3,838 for the specialist and generalist models, respectively. Planning activities had the greatest impact on costs for both models ($2,450 and $1,841 for the specialist and generalist models, respectively). This was followed by contracted services for training and technical assistance ($1,792 and $1,216 for the specialist and generalist models, respectively). The average cost of staff time spent in training was similar across the two models (approximately $770). Overall, more resources were devoted to planning activities in specialist sites, making this model of delivery slightly more costly than the generalist model, largely due to its increased complexity.

## Conclusions

The initial resource investment for providers to implement SBI should not be ignored as these costs may present obstacles toward implementation. The level of resources depend on the delivery model and its integration in current practice.

